# The role of axonal Kv1 channels in CA3 pyramidal cell excitability

**DOI:** 10.1038/s41598-017-00388-1

**Published:** 2017-03-22

**Authors:** Sylvain Rama, Mickael Zbili, Aurélie Fékété, Mónica Tapia, Maria José Benitez, Norah Boumedine, Juan José Garrido, Dominique Debanne

**Affiliations:** 1 UNIS, INSERM UMR_S 1072, Aix-Marseille Université, 13015 Marseille, France; 2 Instituto Cajal, CSIC, Madrid, 28002 Spain; 3 Universidad Autónoma de Madrid, 28049 Madrid, Spain

## Abstract

Axonal ion channels control spike initiation and propagation along the axon and determine action potential waveform. We show here that functional suppression of axonal Kv1 channels with local puff of dendrotoxin (DTx), laser or mechanical axotomy significantly increased excitability measured in the cell body. Importantly, the functional effect of DTx puffing or axotomy was not limited to the axon initial segment but was also seen on axon collaterals. In contrast, no effects were observed when DTx was puffed on single apical dendrites or after single dendrotomy. A simple model with Kv1 located in the axon reproduced the experimental observations and showed that the distance at which the effects of axon collateral cuts are seen depends on the axon space constant. In conclusion, Kv1 channels located in the axon proper greatly participate in intrinsic excitability of CA3 pyramidal neurons. This finding stresses the importance of the axonal compartment in the regulation of intrinsic neuronal excitability.

## Introduction

Ion channels in the axon determine both the generation of the action potential (AP) in the axon initial segment (AIS) and its conduction along the axon proper to the presynaptic terminals^1^. Voltage-gated ion channels in the axon also control the spike waveform and thus, voltage change in the soma determines output strength^2–8^.

Among voltage-gated channels, Kv1 channels play a unique role. They are responsible for the fast-activating, slowly-inactivating D-type current which is broadly expressed in neurons of the central nervous system including CA1 and CA3 pyramidal neurons^9, 10^, L5 and L2/3 pyramidal cells^11, 12^ and parvalbumin (PV)-positive fast-spiking interneurons^13, 14^. Given that *I*_D_ is activated at voltages near AP threshold, it has potentially a critical role in determining the transformation from synaptic input into AP output. In fact, the D-type current plays an important role in controlling AP shape and threshold^4, 11, 15^, synaptic strength^4, 8, 15^, spike-time precision^16^ and synaptic delay^17^. It also enhances the fidelity of neurotransmission^18^. In addition, several forms of long-lasting modulation of intrinsic excitability result from the regulation of Kv1 channels^16, 19, 20^.

The location of Kv1 channels responsible for the modulation of neuronal excitability in the cell body is not precisely known. Depending on studies it can vary from distal dendrites^21^, to proximal dendrites^22^, AIS and axon^4, 13, 23, 24^. We therefore determined here the location of functional Kv1 channels in CA3 pyramidal cells that display a strong D-type current^16^. Using a combination of pharmacological tools in parallel with mechanical or laser axotomy, we show that Kv1.1 channels in the AIS and in the axon trunk regulate the intrinsic excitability of CA3 pyramidal cells measured in the soma.

## Results

### Kv1.1 channels in the AIS and the axon proper of hippocampal cells

**Figure 1:**
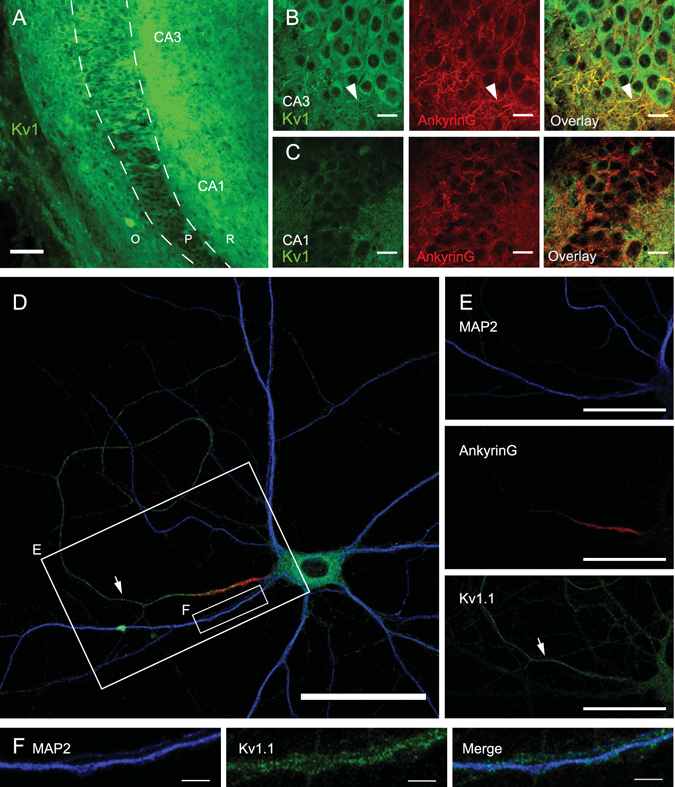
Kv1.1 channels are enriched in AIS area of CA3 pyramidal cells. (**A**) General view of an organotypic culture immunostained for Kv1.1 channels. Note the lack of Kv1.1 immunostaining in CA1 and the strong staining in CA2 and CA3. O, P, R: Oriens, Pyramidale & Radiatum areas. (**B**) Staining of CA3 area with Kv1.1 antibodies (left), ankyrin G antibodies (middle) and superimposition of both (right). Note the strong staining of cell soma with Kv1.1 antibodies and the staining of an extension from soma (left panel, arrowhead). Ankyrin G antibodies stain the AIS (middle panel, arrowhead), co-localised with Kv1.1 staining (right panel, arrowhead). (**C**) Representative views of CA1 area stained with Kv1.1 antibodies (left), Ankyrin G antibodies (middle) and superposition of both (right). Kv1.1 antibodies do not stain any particular structures and are not present in the cell bodies of pyramidal cells (left). Superposition of Kv1.1 and Ankyrin G staining does not show any co-localisation (right). (**D**) 19 DIV hippocampal neuron immunostained with the somatodendritic marker MAP2 (blue), the AIS marker AnkyrinG (red) and Kv1.1 (green) revealing a strong staining in the axon (arrowhead). (**E**) Detail of the area showed in (**D**). Note the Kv1.1 staining all along the axon (arrows). (**F**) Detail on a dendrite showed in (**D**), to show the lack of colocalization between MAP2 (left) and Kv1.1 (middle) staining. Right: composite image with both stainings. Scale bars: 20 µm (**B,C**), 50 µm (**A,D,E**) 0.2 µm (**F**).

We first determined the location of Kv1.1 channels in CA3 neurons from organotypic slice cultures. Slices were cultured for 8–10 days *in vitro* before being re-sectioned at 14 µm with a cryostat and processed for immunohistochemistry (see Experimental Procedures). Kv1.1 immunostaining was observed in both the cell body and the AIS identified by Ankyrin G immunostaining in CA3 neurons (Fig. 1A and B). The length of the AIS in CA3 pyramidal cells was found to be comparable with values found in acute slices^25^ (55.9 ± 0.1 µm, n = 34 AIS). Interestingly, CA1 pyramidal cells expressed no Kv1.1 immunostaining (Fig. 1C). This lack of Kv1.1 labelling in CA1 pyramidal cells at this relatively early stage of development (slices cut at P7 and 8–10 days of development *in vitro*) can easily be explained by the fact that *I*_D_ is mainly expressed in CA1 after P21^26^. Moreover, Kv1.1 channels are expressed at P6 in the area CA3^27^.

To better identify the precise location of Kv1.1 channels in the subcellular compartment of the neurons, we used hippocampal neurons from dissociated cultures (see Experimental procedures). Kv1.1 immunostaining was not only limited to the AIS revealed by immunostaining of Ankyrin G but clearly extended up to the end of the axon collaterals (Fig. 1D). While the soma displayed an intracellular staining, Kv1.1 immunostaining was found to be located in the AIS and all along the axon (Fig. 1E, arrows). The Kv1.1 staining observed near the dendrites corresponded to axons running along dendrites (Fig. 1F). Next, we have examined whether Kv1.2 were also present on the axon or dendrites of hippocampal cells. In contrast with the labeling of Kv1.1 channels, the labeling obtained with Kv1.2 was found to be much lighter (Supplementary Figure 1). We conclude that Kv1.1 channels are located all along the axon of hippocampal neurons.

### Axonal but not dendritic Kv1.1 channel blockade increases somatic excitability

**Figure 2:**
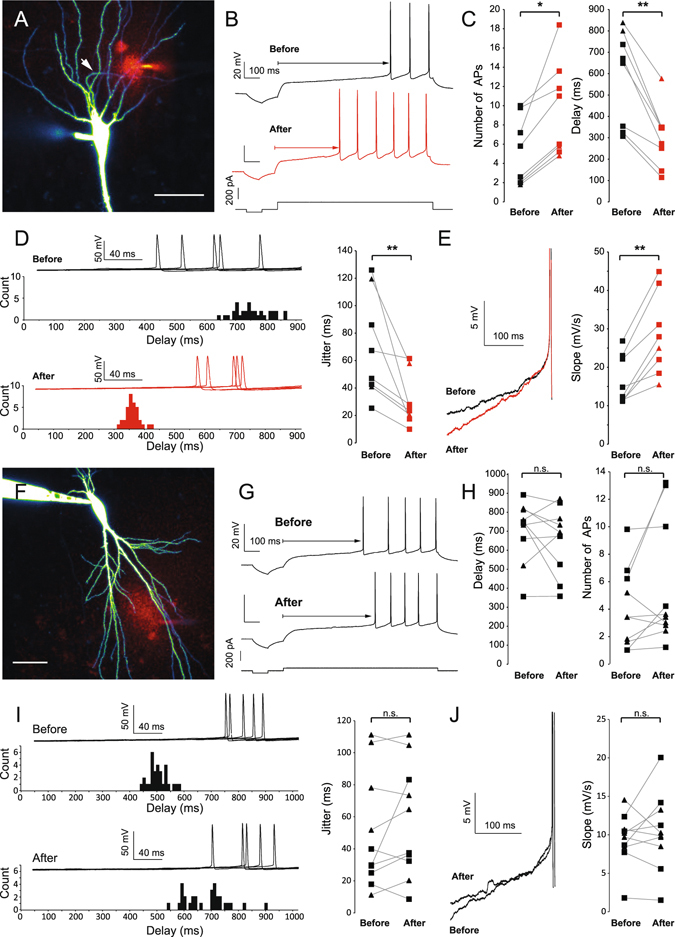
DTx puffs on the axon increase somatic excitability. (**A**) Puff of DTx-K and Alexa 594 on the axon of a CA3 pyramidal neuron filled with Alexa 488. Arrow: axon. Scale Bar: 50 µm. (**B**) Example of spike discharge before (black) and after (red) 40 puffs of DTx-K on the AIS. Note the increased number of APs and the decreased delay to the first spike. (**C**) Pooled data of the impact of DTx-K and DTx-I puffs on the axon (0–150 µm from the soma). Stars: Wilcoxon test, p < 0.05, n = 8. (**D**) Effect of DTx puffs on the jitter of the first AP. Stars: Wilcoxon test, p < 0.05, n = 8. (**E**) Effect of DTx puffs on the depolarizing slope before the first AP (traces aligned on the spike threshold). Stars: Wilcoxon test, p < 0.05, n = 8. (**F**) Puff of DTx-K and Alexa 594 on the dendrites of a CA3 pyramidal neuron filled with Alexa 488. Scale Bar: 50 µm. (**G**) Example of spike discharge before (top) and after (bottom) 40 puffs of DTx-K on the dendrites. (**H**) Pooled data of the impact of DTx-K and DTx-I puffs on the dendrites (0–150 µm from the soma). (**I**) Effect of dendritic puff of DTx on the jitter of the first spike. (**J**) Effect of DTx puffs on the depolarizing slope before the first AP. For panels C, D, E, H, I & J: squares: puffs of DTx-K, triangles: puffs of DTx-I.

**Figure 3:**
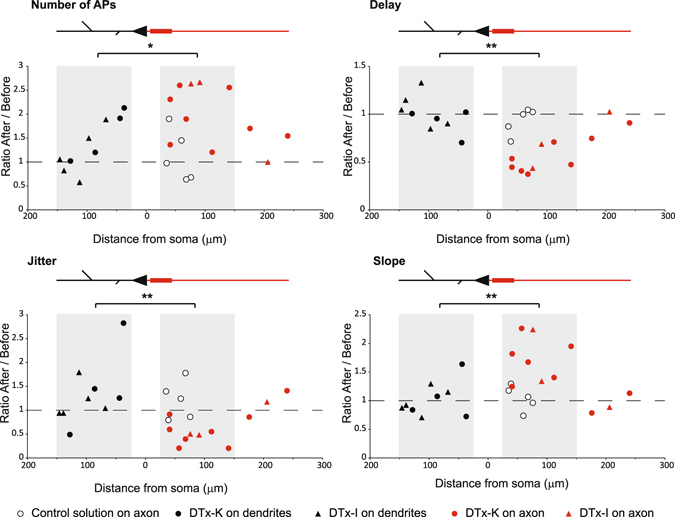
Effect of puffing DTx-K or DTx-I on global excitability according to the distance from the soma. Full set of experiments, showing the relative effect of DTx-K or DTx-I on the delay, number of APs, jitter and slope and according to the distance from soma, on axon or dendrites of CA3 pyramidal cells. White circles: puff of control extracellular solution on the axon; black circles: puff of DTx-K on dendrites; red circles: puff of DTx-K on axon; black triangles: puff of DTx-I on dendrite; red triangles: puff of DTx-I on axon. Note the maximum effect of DTx on the excitability around 50 µm on the axon, which coincides with AIS position. Grey areas: experiments showed in Fig. 2. Star: Mann-Whitney test, p < 0.05 (n = 9 for dendritic puffs and n = 8 for axonal puffs); Double star: Mann-Whitney test, p < 0.01 (n = 9 for dendritic puffs and n = 8 for axonal puffs). All puffs of control solution on axon are significantly different from DTx puffs on axon (Mann-Whitney tests, all p < 0.05 (n = 5 for axonal puffs of control solution and n = 8 for axonal puffs of DTx)).

To assess the contribution of axonal Kv1 channels on intrinsic neuronal excitability, we monitored the excitability of CA3 pyramidal cells before and after puffing the specific Kv1.1 channel blocker, DTx-K or the broader spectrum Kv1 channel blocker, DTx-I. CA3 pyramidal cells were recorded with a pipette containing Alexa 488 (50 μM) to reveal their morphology (Fig. 2A). Long depolarizing current pulses were injected in the soma to elicit a few spikes in control conditions (Fig. 2B). The extent of the DTx application was visualized with addition of Alexa 594 (50 μM) to the pipette medium (see Experimental Procedures). Puffing DTx on the proximal part of the axon (0–150 μm from the soma) roughly increased the excitability by a factor 2. The AP number increased from 5.2 ± 1.2 in control to 9.5 ± 1.7 after application of DTx-K & I, n = 8, p < 0.05 Wilcoxon) and the delay to the first AP was reduced (from 585 ± 78 ms vs. 300 ± 51 ms, n = 8, p < 0.01, Wilcoxon; Fig. 2C). In parallel, the jitter of the first AP decreased (from 69 ± 13 ms to 31 ± 6 ms, n = 8, p < 0.01, Wilcoxon; Fig. 2D) and the depolarizing slope measured 100 ms before the first AP increased (from 16 ± 2 mV/s to 28 ± 3 mV/s, n = 8, p < 0.01, Wilcoxon; Fig. 2E). In contrast, puffing DTx on the dendrites (n = 10) (Fig. 2F–J) or puffing ACSF on the AIS (see Fig. 3 for statistics) produced no change in excitability neither on the delay of the first spike, the jitter of the first spike, nor on the depolarizing slope before the first spike.

In order to characterize the spatial extent of DTx application, the effects were plotted against the location of the puff (Fig. 3). The strongest effect of DTx was observed on the axon (at ~50 μm from the soma), i.e. corresponding to the location of the AIS. Interestingly, the effects decreased with the distance but were still high far away from the AIS area. The slight increase in excitability observed when DTx was puffed on proximal dendrites (Fig. 3) might be due to the relatively large diffusion area of DTx (estimated to be 48 ± 2 µm, n = 33) that may possibly inhibit Kv1 channels located on both the soma and the AIS.

### Axotomy but not dendrotomy increases excitability recorded at the soma

**Figure 4:**
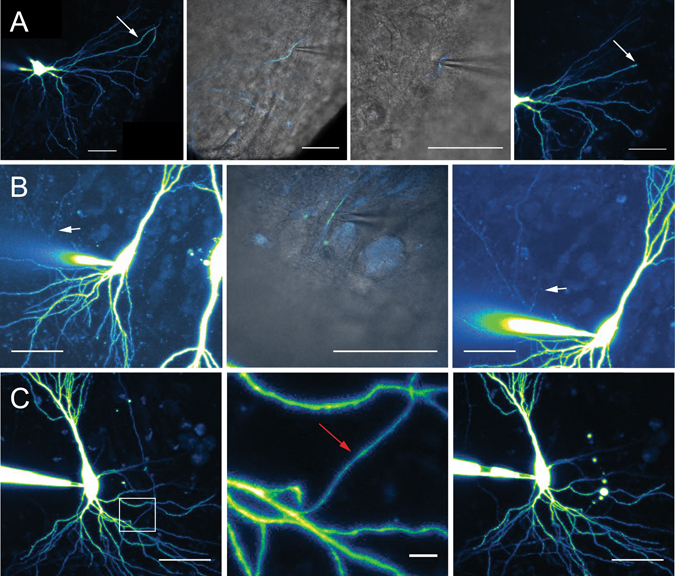
Methods of dendritic and axonal resection and burning. (**A**) Typical dendrite resection. Left panel: after filling a CA3 pyramidal neuron with Alexa 488 50 µM, dendritic branches could be imaged from apical or basal side of the neuron. Arrow: dendritic branch chosen to be cut. Middle left panel: by using the transmitted light PMT, a second pipette was precisely placed under the selected dendritic branch. Middle right panel: cautiously moving the pipette, it was possible to pull out the dendrite from the slice. Right panel: a brief and strong upward movement results in the dendrite resection, at the point chosen before. Note the absence of Alexa 488 fluorescence downstream of the section. Scale bars: 20 µm. (**B**) Same protocol applied to axonal branches. Left panel: a collateral crossing the pyramidal layer was chosen. Middle panel: pipette pulling out the axonal branch. Right panel: after section, no fluorescence is visible downstream of the section point. Scale bars: 20 µm. (**C**) Laser burning of an axon collateral. Left panel: confocal acquisition of a CA3 pyramidal neuron. Middle panel: enlargement of the white box showed in the left panel. The axon is clearly identified as a thin, aspiny process. Red arrow: chosen burning point. Right panel: after cautious burning in line-scan mode, membrane bubbles develop downstream of the burning point, and Alexa 488 staining disappears. No effect can be seen upstream the burning point. Scale bars: 20, 5 and 20 µm.

**Figure 5:**
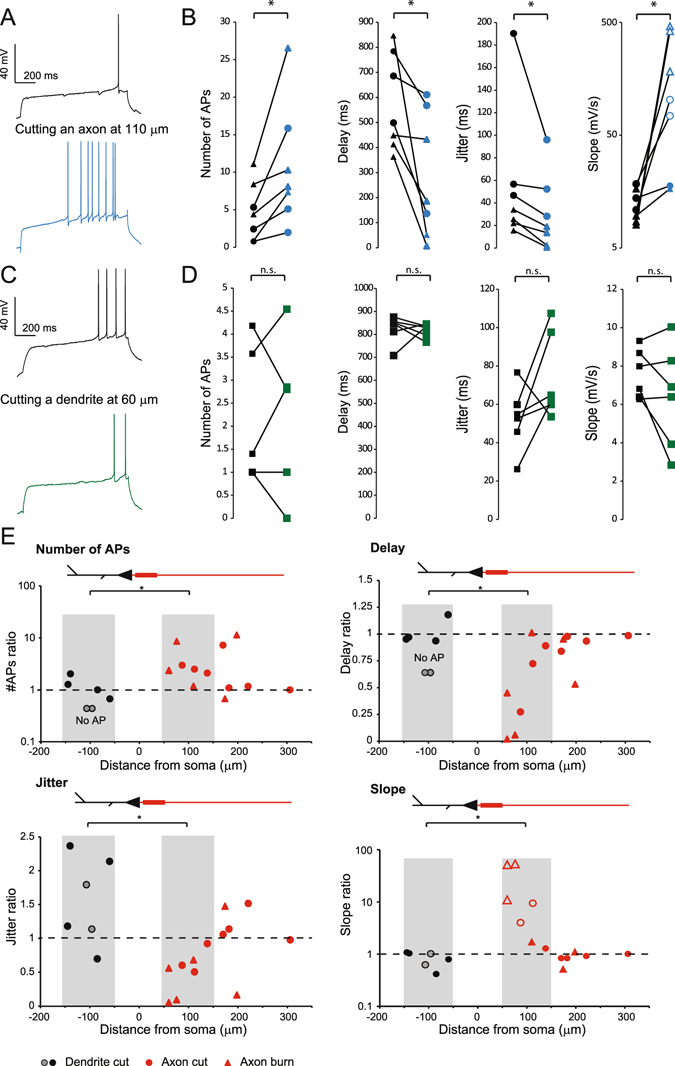
Excitability is increased by axon section but not dendrite section. (**A**) Typical example showing the excitability before (top) and after (bottom) axon resection or burning. (**B**) Pooled data of the effects of axon resection or burning on the spike number, the delay to the first spike, the jitter and the depolarizing slope (n = 7) Star: Wilcoxon test, p < 0.05. Triangle: burning, Circles: cutting. Note that the empty circles in the slope panel correspond to slopes measured 20 ms (and not 100 ms) before the first spike because the delay was too short. (**C**) Typical example showing the excitability before (top) and after (bottom) dendrite resection. (**D**) Pooled data of the effects of dendrotomy on the spike number, the delay to the first spike, the jitter and the depolarizing slope (n = 6). (**E**) Relative effects of dendrotomy and axotomy as a function of the distance from soma, on AP number, delay, jitter and slope. Black circles: dendrite cut; grey circles: dendrite cuts resulting in a disappearance of APs during the depolarizing step. Red circles: axon cut; Red triangles: axon burn. Grey areas on each graph correspond to the experiments used for statistics in Fig. 5B and D.

Because of the spreading geometry of the axon, puffing DTx on proximal dendrites may well affect the axonal compartment. To circumscribe this possible issue, we sectioned axon or dendrite collaterals and studied their impact on neuronal excitability^28^. CA3 neurons were recorded with a pipette containing Alexa 488 (50 μm) to visualize the axonal arborisation. To cut axon or dendrite collaterals, a second pipette was placed below the collateral at 60 to 306 µm from the cell body and pulled up out of the slice (Fig. 4A and B). The efficiency of the section was confirmed by the disappearance of fluorescence downstream of the cut. Cutting axon collaterals was impossible near the AIS because it destabilized the recording. To circumvent this problem, we used the laser axotomy technique^29^ in which the axon was continuously illuminated in line scan mode (see Experimental Procedures; Fig. 4C). Cutting axon collateral slightly decreased input resistance (from 135 ± 14 to 130 ± 14 MΩ, n = 14, p < 0.001, Wilcoxon test) but had no significant effect on membrane capacitance (from 240 ± 15 to 237 ± 15 pF, n = 14, p > 0.05, Wilcoxon test). Importantly, it also increased neuronal excitability (from 5 ± 2 to 10 ± 3 spikes, n = 7; Fig. 5A). The first spike latency was found to be reduced (from 577 ± 73 to 285 ± 94 ms, n = 7; Fig. 5B), the slope before the first spike increased from 12.4 ± 1.4 to 181 ± 89 mV/s (n = 7) and the jitter of the first spike decreased from 56 ± 23 to 30 ± 13 ms (n = 7). In contrast, cutting dendrites produced no significant effect on intrinsic excitability (from 2 ± 0.6 to 1.9 ± 0.7 spikes, n = 6), the delay before the first AP (from 824 ± 25 to 818 ± 12 ms, n = 6), the depolarizing slope (from 7 ± 0.5 to 6.3 ± 1.1 mV/s, n = 6) or the jitter of the first AP (from 53 ± 7 to 74 ± 10 ms, n = 6) (Fig. 5C and D). Interestingly, the effects of axonal section were the largest near the AIS and declined with distance (Fig. 5E), reaching the baseline at approximately 200 µm from the soma. We conclude that axotomy beyond the AIS selectively enhances excitability indicating that axonal Kv1.1 channels control excitability measured in the soma.

### A model with Kv1 channels in the axon reproduced the experimental observations

**Figure 6:**
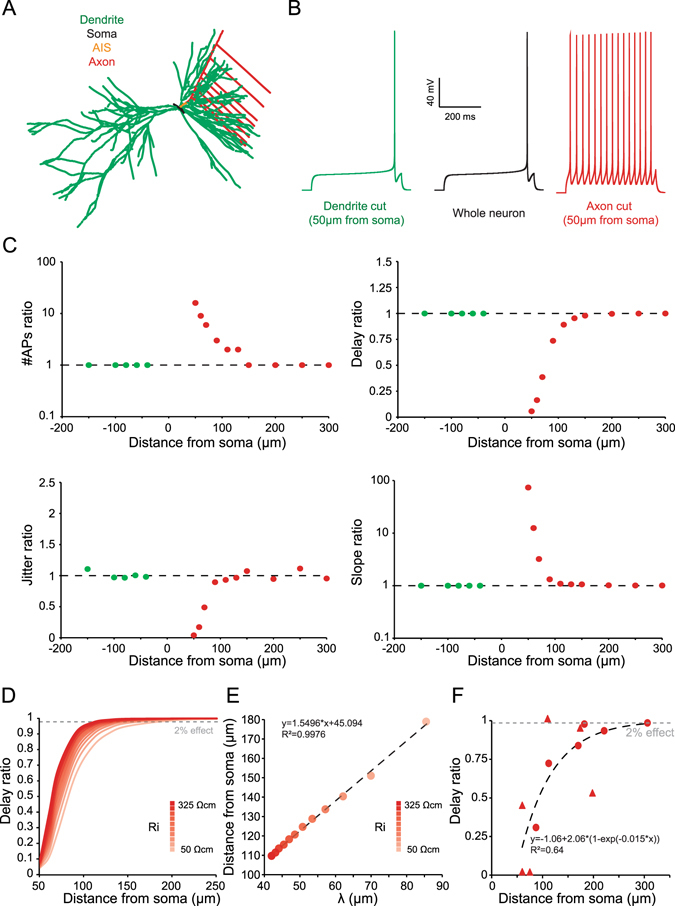
Modelling study of axon and dendrite cuts. (**A**) Drawing of the computational model. (**B**) Effect of axonal or dendritic cut on excitability and first spike delay during a 1 s current injection. Note the increase of AP number and decrease of first spike delay after axonal cut. (**C**) Spatial effect of axonal or dendritic cutting on number of APs, first spike delay, first spike jitter and depolarizing slope before the first spike during a 1 s current injection. Note that the model reproduces the experimental results of Fig. 3D. (**D**) Spatial extent of spike delay decrease by axonal cutting with various intracellular resistivity (R_i_). Note that the larger the R_i_, the shorter the spatial extent. (**E**) Distance of axonal cut to get a 2% spike delay decrease as a function of space constant (λ). Note the linear relationship. (**F**) Fit of experimental data of delay reduction by axonal cutting (from Fig. 5D).

To further understand the role of axonal Kv1 channels in the control of intrinsic excitability, we used a modelling approach (Fig. 6A). All the compartments contained passive conductance, voltage-dependent Na^+^ and delayed rectifier K^+^ channels (see Experimental Procedures). The D-type conductance was only inserted in the AIS and the axon. All parameters were measured in the soma, to mimic our experimental observations. As in the experiments, removing the axon after the AIS increased the excitability of the model whereas removing a dendrite had no effect (Fig. 6B). The model reproduced the distance dependent effect of axotomy and dendrotomy (Fig. 6C, see Fig. 6E for comparison). The axon cut entailed an increase of the spike number, a decrease of the first spike delay, a decrease of the jitter and an increase of the depolarizing slope before the first AP (red dots in Fig. 6C). Moreover, as observed in our experiments these effects depended on the distance were we performed the simulated axotomy. In contrast, dendrotomy had no effect regardless of the cutting distance (green dots in Fig. 6C).

In order to characterize the distance dependence of axonal removal, we plotted the effect on the delay to the first spike as a function of the distance of the axon collateral cut with varying intracellular resistivity (R_i_). R_i_ determines the axonal space constant (λ), in an inverse proportional way (λ ∝ 1/√R_i_). A distal axonal cut (150 µm) had an impact on the first spike delay only when the R_i_ was small (150 Ωcm or less) but not when it was large (300 Ωcm) (Fig. 6D).

We set as the threshold for detecting an effect on the delay, the cutting distance that produced 2% decrease. This critical distance was found to vary as a function of R_i_ (on the dotted line in Fig. 6D) and a linear relationship between λ and the threshold cutting distance was found (Fig. 6E). By fitting our experimental results, the axonal cutting distance leading to a 2% decrease in the delay was found to be 297 µm (Fig. 6F) and the λ was estimated to be ~163 µm. This value is consistent with previous findings showing that Kv1-dependent enlarging of AP has a space constant of 145 μm in CA3 neurons^30^. In conclusion, the model reproduced the effects of axon or dendrite cuts and it shows that the critical distance at which the cuts have an effect is determined by cable properties.

### Relative contribution of axonal versus somato-dendritic Kv1 channels to CA3 excitability

Even if no obvious impact was observed on intrinsic excitability upon cutting a single dendrite, one cannot exclude the possibility that a small amount of functional Kv1 channels are present in the somato-dendritic compartment. As the dendritic surface is large, sparsely distributed Kv1 channels could be well sufficient to regulate intrinsic excitability recorded in the soma. To investigate this possibility, Kv1 channels were successively inhibited in the different compartments of the neuron (Supplementary Figure 2A). We started by sectioning the axon just after the AIS (at 50–90 μm from soma) to inhibit Kv1 channels in the axon proper. Then we puffed DTx-K on the AIS. Finally, DTx-K was bath-applied to inhibit Kv1.1 channels located in the soma and the dendrites. Intrinsic excitability was monitored in each step of the experiment (Supplementary Figure 2B). Following bath application of DTx-K, intrinsic excitability was found to be increased: the number of evoked action potentials increased from 7 ± 2 to 13 ± 3 spikes, n = 7; p < 0.05, Wilcoxon test). The first spike latency was found to be reduced (from 157 ± 73 to 25 ± 5 ms, n = 7; p < 0.05, Wilcoxon test), the jitter of the first spike decreased (from 23 ± 11 to 2.5 ± 1 ms; n = 7; p < 0.05, Wilcoxon test), but the slope before the first spike did not change (from 760 ± 170 to 831 ± 140 mV/s; n = 7; p > 0.05, Wilcoxon test). To compare the contribution of Kv1 channels in the axon proper and in the somato-dendritic compartment to intrinsic excitability, we normalized the different parameters measured in the situations “*Axon cut*” and “*DTx-K in the bath*” with the “*Control*” situation, and with the “*DTx-K puff on AIS*” situation, respectively (Supplementary Figure 2C). Except for the slope (p < 0.05, Mann-Whitney test), the two conditions were not statistically different. This normalization shows that cutting the axon below the AIS is roughly the same than inhibiting Kv1.1 channels on all somatic and dendritic compartments.

## Discussion

The present study demonstrates that Kv1.1 channels located in the axon determine the excitability of CA3 pyramidal neurons. Using a combination of techniques including immunohistochemical labelling, targeted axotomy, focal block of Kv1.1 channels and modelling, it was found that Kv1.1 channels are essentially located in the AIS and the axon and that their functional removal beyond the AIS significantly alter intrinsic excitability and spike firing properties measured in the soma. Moreover, it underlines the importance of preserving the whole axonal arborisation to study the excitability of central neurons.

### Axonal Kv1 channels determine intrinsic excitability in CA3 pyramidal cells

We establish here that axonal Kv1 channels determine excitability measured in the cell body of CA3 pyramidal neurons. The axonal location of Kv1 channels is relatively well established. In neocortical pyramidal cells, functional Kv1 channels are highly expressed in the AIS and beyond, in the axon proper^4^. In fast spiking basket-cell interneuron, DTx applied focally has an effect only on the axon initial segment but not on the cell body^13^. However, in these studies it was not clear whether the excitability of the cell measured in the cell body was influenced by Kv1 channels located farther than the AIS. We show here that axonal Kv1 channels located beyond the AIS control the excitability of CA3 pyramidal cells recorded in the soma. The elimination of functional Kv1 channels in the axon was made through 2 techniques. In a first step, focal puffs of DTx-K or DTx-I were found to increase excitability of pyramidal cells when they were applied on the axon but not on the dendrites. As similar effects were obtained with the two toxins, we conclude that the increase in excitability is mediated by inhibition of axonal Kv1.1 channels. These changes in excitability were associated with a reduced delay to the first spike, a hallmark of the action of the Kv1-mediated D-type current. In a second step, axon collaterals were cut mechanically or after high intensity laser illumination. Here again, excitability was significantly increased after physical elimination of axon collaterals but not after cutting dendrites. Interestingly, axon cuts up to 150–200 µm from the soma (i.e. ~90–140 µm from the end of the AIS) induced a significant elevation in excitability associated with a reduced delay to the first spike, a reduced jitter and an increase in the depolarizing slope before the spike. The increase in excitability observed experimentally after axotomy was not the result of the deterioration of the recording. The membrane capacitance remained unchanged in these experiments. Similar changes in excitability were observed after local application of Kv1.1 channel blocker and the experimental observations were reproduced in the model.

### Spike jitter is determined by axonal Kv1.1 channels

Kv1 channel is a primary determinant of spike-time precision in CA3 pyramidal cells^16^. In fact, the Kv1-mediated D-type current is thought to reduce spike-time precision by reducing the rate of depolarization before the spike^16^, thus introducing a larger jitter in spike-timing^31^. We show here that blocking axonal Kv1.1 channels with local puffs of DTx increased spike-time precision and the rate of depolarization before the spike. Similar results were observed following mechanical or laser axotomy of collaterals. However, no improvement of spike-time precision was observed after focal application of DTx in the dendrites or after dendrotomy. The modification observed after focal blockade of Kv1 channels with DTx is very similar to those induced by a global block with bath application of DTx^16^, suggesting that all the effect is mediated by Kv1 channels located in the axon.

### The axon as a regulator of intrinsic excitability

Our study shows that ion channels located in the axon are major determinants of the whole neuronal excitability. Thus, integration of neuronal information is not limited to the somato-dendritic region of the neuron as initially thought^32^. Rather, in the case of CA3 pyramidal cells, it extends up to the first 150–200 µm of their axon. Our modelling study indicates that the impact axonal channels have on excitability is mainly determined by the axonal space constant. The fact that axonal ion channels determine firing properties had been previously reported in the case of neocortical neurons in which Na^+^ channels located at ~120 µm from the soma were responsible for intrinsic bursting^29^. Moreover, we could estimate that the potency of axonal Kv1 channels in the axon proper is roughly the same than for Kv1 channels located in the whole somato-dendritic area. The finding that distal axonal Kv1 channels control excitability of CA3 pyramidal cells raises some concerns about studies conducted in acute slices. Because axons might be severed at different distance from one cell to the other, it might introduce important variability in the measures of intrinsic excitability.

In conclusion, the axon of CA3 pyramidal cells cannot be considered anymore as a pure conduction cable without any interaction with the cell body. Rather, the axon proper at the AIS and beyond the AIS appears to be an essential determinant of neuronal excitability.

## Experimental procedures

### Organotypic cultures of rat hippocampus

Hippocampal slice cultures were prepared as described previously^33^. All experiments were carried out according to the European and Institutional guidelines for the care and use of laboratory animals (Council Directive 86/609/EEC and French National Research Council) and approved by the local health authority (#D1305508, Préfecture des Bouches-du-Rhône, Marseille). Postnatal day 7–8 Wistar rats were deeply anesthetized by intraperitoneal injection of chloral hydrate, the brain removed and each hippocampus dissected. Hippocampal slices (350 µm) were placed on 20 mm latex membranes (Millicell) inserted into 35 mm petri dishes containing 1 ml of culture medium and maintained for up to 21 days in an incubator at 34 °C, 95% O_2_–5% CO_2_. The culture medium contained (in ml) 25 MEM, 12.5 HBSS, 12.5 horse serum, 0.5 penicillin/streptomycin, 0.8 glucose (1 M), 0.1 ascorbic acid (1 mg/ml), 0.4 HEPES (1 M), 0.5 B27, 8.95 sterile H_2_O. To limit glial proliferation, 5 µM Ara-C was added to the culture medium.

### Hippocampal neurons culture

Mouse hippocampal neurons were prepared as previously described^34^. Neurons were obtained from E17 mouse hippocampi, which were incubated in a 0.25% trypsin solution in Ca^2+^/Mg^2+^ free Hank’s buffered salt solution (HBSS) and dissociated using fire polished Pasteur pipettes. The cells were plated on polylysine-coated coverslips (1 mg/mL) at a density of 5000 cells/cm^2^ for 2 h in plating medium (minimum essential medium [MEM], 10% horse serum, 0.6% glucose, Glutamax-I and antibiotics). Then, coverslips were inverted and transferred to culture dishes containing astrocytes. Astrocytes medium was replaced by neuronal culture medium 24 h before (Neurobasal medium, B27 supplement, Glutamax-I). To avoid contact between neurons and astrocytes paraffin beads were placed on coverslips before neuronal plating. 5 μM 1-β-D-arabinofuranosylcytosine (AraC) was added to avoid glial proliferation. Neurons were cultured for 19–23 DIV before fixation.

### Immunohistochemistry

Organotypic slices were fixed with paraformaldehyde 4% during 15 min, washed 3 times with PBS and then put in a 30% sucrose solution overnight. Each culture was embedded with Tissue-Tek OCT Compound (VWR) and sliced at 14 µm with a cryostat. Immunostaining of Kv1.1 channels was performed. Double labeling of ankyrin G (rabbit, 1/500, A30, gift of F Castets) and Kv1.1 (extracellular epitope, mouse, 1/50, NeuroMAB). Secondary antibodies were raised in donkey and tagged with distinct fluorophores (anti-mouse Alexa 488; anti-rabbit Alexa 594). Confocal image acquisition was performed on a Leica TCS SP2 laser scanning microscope (Leica Microsystem, Heidelberg, Germany) using 488 nm band of an Ar laser for excitation of Alexa-Fluor 488 (spectral detection: 492–544 nm), and 545 nm laser diode for the excitation of Alexa 594 (spectral detection: 610–650 nm).

Immunofluorescence in cultured hippocampal neurons was performed as follows. Coverslips were fixed in PFA 4%/Sucrose 4% for 20 minutes at RT, treated for 10 min with 50 mM NH_4_Cl and incubated in blocking buffer (0.22% gelatin, 0.1% Triton X-100 in PBS) for 30 min, before incubation with primary antibodies for 1 h at RT in blocking buffer. The primary antibodies used were: chicken anti-MAP2 (1/10000, Abcam), mouse anti-ankyrinG (1/100, NeuroMab N106/36, IgG2a), mouse anti-Kv1.1 (extracellular epitope, 1/100, NeuroMab K36/15, IgG2b) and mouse anti-Kv1.2 (1/100, NeuroMab K14/16, IgG2b). The secondary antibodies used were goat anti-mouse IgG2a-Alexa-Fluor-594, IgG2b-Alexa-Fluor-488 and anti-chicken-Alexa-Fluor-647 (1/1000). Images were acquired in a Leica TCS SP5 laser scanning microscope (Leica Microsystem).

### Electrophysiological recordings and data analysis

CA3 pyramidal neurons were whole-cell recorded in organotypic cultures. The external solution contained (mM): 125 NaCl, 26 NaHCO_3_, 3 CaCl_2_, 2.5 KCl, 2 MgCl_2_, 0.8 NaH_2_PO_4_ and 10 D-glucose, and was equilibrated with 95% O_2_−5% CO_2_. Patch pipettes (5–10 MΩ) were filled with a solution containing (in mM): K-gluconate 120; KCl 20; HEPES 10; EGTA 0.5; MgCl_2_ 2; Na_2_ATP 2; NaGTP 0.3 (pH 7.4). All recordings were made at 29 °C in a temperature-controlled recording chamber (Luigs & Neumann, Ratingen, Germany). Neurons were recorded in current clamp with a Multiclamp 700B amplifier (Axon Instruments, Molecular Devices) and were held at resting membrane potential (~−65 mV). The effect of the D-current was monitored by injecting a long (1 s) depolarizing current pulse. The voltage and current signals were low-pass filtered (3 kHz), and acquisition was performed at 10 kHz with pClamp10 (Axon Instruments). Access resistance and capacitance were checked by injecting a small hyperpolarizing current. Data were analyzed with ClampFit (Axon Instruments) or self-developed software written in Labview (National Instrument). Pooled data are presented as mean ± SE and statistical analysis was performed using the Mann-Whitney *U*-test or Wilcoxon rank-signed test.

### Confocal Imaging

CA3 neurons were filled with Alexa 488 50 µM (Invitrogen) for visualizing neuronal morphology. Neurons were recorded for ~10 minutes before imaging with a LSM 710 confocal system (Zeiss). Alexa 488 and 594 were excited with laser source at 488 nm and 543 nm, respectively. The axon was identified by visual clues: a thin, aspiny process emerging from the soma or a proximal dendrite. Axon collaterals emerging at right angle, and presynaptic boutons could be identified at ~150 µm from the soma.

### Puff applications

Localized puffs of Kv1 channel blocker were applied on the axon or a dendrite of the recorded neuron with a patch-pipette filled with a solution containing the extracellular saline with 50 µM Alexa 594 (Invitrogen) and 100 nM DTx-K or DTx-I (Sigma). Short puffs (20–50 ms, 5 psi) were emitted every 10 s by a Toohey-Spritzer pressure system (Toohey Company). The fluorescence signals produced by puffs of Alexa 594 were acquired over episodes of 5 puffs, averaged and fitted with a Gaussian curve to estimate the area of DTx application. The axon was visually identified as the only neuronal process devoid of spines, with constant diameter, emerging from the soma or from a primary dendrite near the cell body.

### Axon and dendrite cuts

For cutting dendrite or axon collaterals, neurons were filled with Alexa 488 and a patch-pipette was positioned below the collateral. By gently pulling up the pipette, the axon collateral was pulled out of the slice and then cut with a swift upward movement of the micro-manipulator. Axons or dendrites were cut in the same way. However, for cutting axon collaterals that are either too proximal or entangled with dendrite arbor we used the laser-burning technique. The axotomy was performed in line scan mode and the chosen area was illuminated with medium power laser for 1–2 minutes. Laser illumination was stopped as soon as a slight depolarization of the membrane could be seen. The neuron was let recovering for 3–5 min before checking axon morphology. Successful axotomy was characterized by the presence of membrane swellings and the disappearance of Alexa 488 staining downstream of the illumination.

### Modeling

A multi-compartment model was simulated with Neuron 7.2. The diameter and length of each compartment is provided in Supplemental Table 1. The passive electrical properties C_m_ and R_i_ were set to 1.41 µF/cm^2^ and 80 Ωcm, respectively, uniformly throughout all compartments. All simulations were run with 10 µs time steps and the nominal temperature of simulations was 28 °C. The voltage-dependence of activation and inactivation of a Hodgkin-Huxley based conductance models (g_Na_, g_KDR_, g_Kv1_) are given as follows:

1 $${g}_{Na}={G}_{Na}{}^{\ast }\,{m}^{3}{}^{\ast }\,h$$

2 $${g}_{KDR}={G}_{KDR}{}^{\ast }\,n$$

3 $${g}_{Kv1}={G}_{Kv{1}}{}^{\ast }\,p{}^{\ast }\,k$$

where *m, n* and *p* are dynamic activation variables, *h* and *k* are dynamic inactivation variables. They evolve according to the following differential equations (adapted from^35^ for g_Na_^36^; for K_DR_^37, 38^; for K_v_1):

4 $${\rm{d}}{\rm{m}}/{\rm{d}}{\rm{t}}=({{\rm{m}}}_{{\rm{\infty }}}-{\rm{m}})/{{\rm{m}}}_{\zeta }\,\,with\,{{\rm{m}}}_{\zeta }=0.1$$

5 $${{\rm{m}}}_{{\rm{\infty }}}=1/(1+{{\rm{e}}}^{(0.094{}^{\ast }(-40-{\rm{V}}))})$$

6 $${\rm{d}}{\rm{h}}/{\rm{d}}{\rm{t}}=({{\rm{h}}}_{{\rm{\infty }}}-{\rm{h}})/{{\rm{h}}}_{\zeta }\,\,with\,{{\rm{h}}}_{\zeta }=0.5$$

7 $${{\rm{h}}}_{{\rm{\infty }}}=1/(1+{{\rm{e}}}^{(-0.09{}^{\ast }(-64-{\rm{V}}))})$$

8 $${\rm{d}}{\rm{n}}/{\rm{d}}{\rm{t}}=({{\rm{n}}}_{{\rm{\infty }}}-{\rm{n}})/{{\rm{n}}}_{\zeta }\,\,with\,{{\rm{n}}}_{\zeta }=10$$

9 $${{\rm{n}}}_{{\rm{\infty }}}=1/(1+{{\rm{e}}}^{(0.114{}^{\ast }(13-{\rm{V}}))})$$

10 $${\rm{d}}{\rm{p}}/{\rm{d}}{\rm{t}}=({{\rm{p}}}_{{\rm{\infty }}}-{\rm{p}})/{{\rm{p}}}_{\zeta }\,\,with\,{{\rm{p}}}_{\zeta }=1$$

11 $${{\rm{p}}}_{{\rm{\infty }}}=1/(1+{{\rm{e}}}^{(0.09{}^{\ast }(-43-{\rm{V}}))})$$

12 $${\rm{d}}{\rm{k}}/{\rm{d}}{\rm{t}}=({{\rm{k}}}_{{\rm{\infty }}}-{\rm{k}})/{{\rm{k}}}_{\zeta }\,\,with\,{{\rm{k}}}_{\zeta }=2000$$

13 $${{\rm{k}}}_{{\rm{\infty }}}=1/(1+{{\rm{e}}}^{(-0.18{}^{\ast }(-63-{\rm{V}}))})$$

where V is the membrane potential of the simulated neuron. The equilibrium potential for Na^+^, K^+^ and passive channels was set to +80 mV, −77 mV and −65 mV respectively. The conductance density is provided in Supplementary Table 1. For jitter simulation, we added a Gaussian noise to the injected current (mean: 0; variance: 0.1 pA^2^). Cutting experiments were modeled by simply reducing the length of the considered neurite to 0.01 µm.

## Electronic supplementary material

Supplementary Figures and Table
