# Supplementary material for: The role of axonal Kv1 channels in CA3 pyramidal cell excitability
Source: Sci Rep. 2017 Mar 22;7:315. doi: 10.1038/s41598-017-00388-1 (PMC5428268; doi:10.1038/s41598-017-00388-1)
Supplement: Supplementary file 1 — Supplementary Figures and Table [file 41598_2017_388_MOESM1_ESM.pdf]

Supplementary Information

The role of axonal Kv1 channels in CA3 pyramidal cell excitability

Sylvain Rama[1], Mickael Zbili[1], Aurélie Fékété[1], Mónica Tapia[2], Maria José Benitez[2,3], Norah Boumedine[1], Juan José Garrido[2] & Dominique Debanne[1,4]

[1] UNIS, INSERM UMR_S 1072, Aix-Marseille Université 13015 Marseille, France

[2] Instituto Cajal, CSIC, Madrid 28002, Spain

[3] Universidad Autónoma de Madrid, 28049 Madrid, Spain

[4] correspondence to dominique.debanne@univ-amu.fr

	Dendrite	Soma	Ais	Main axon	Axon collateral
Length (µm)	10-340	30	50	1000	200
Diameter (µm)	1.1-0.3	30	2	1	1
Membrane capacitance (µF)	1.41	1.41	1.41	1.41	1.41
Passive conductance (S/cm²)	0.00004	0.00004	0.00004	0.00004	0.00004
Ina (S/cm²)	0.002	0.05	0.9	0.22	0.22
Id (S/cm²)	0	0	0.06	0.06	0.06
Ikdr (S/cm²)	0.02	0.03	0.2	0.02	0.02

Table 1 – Morphology and conductance distribution of the model

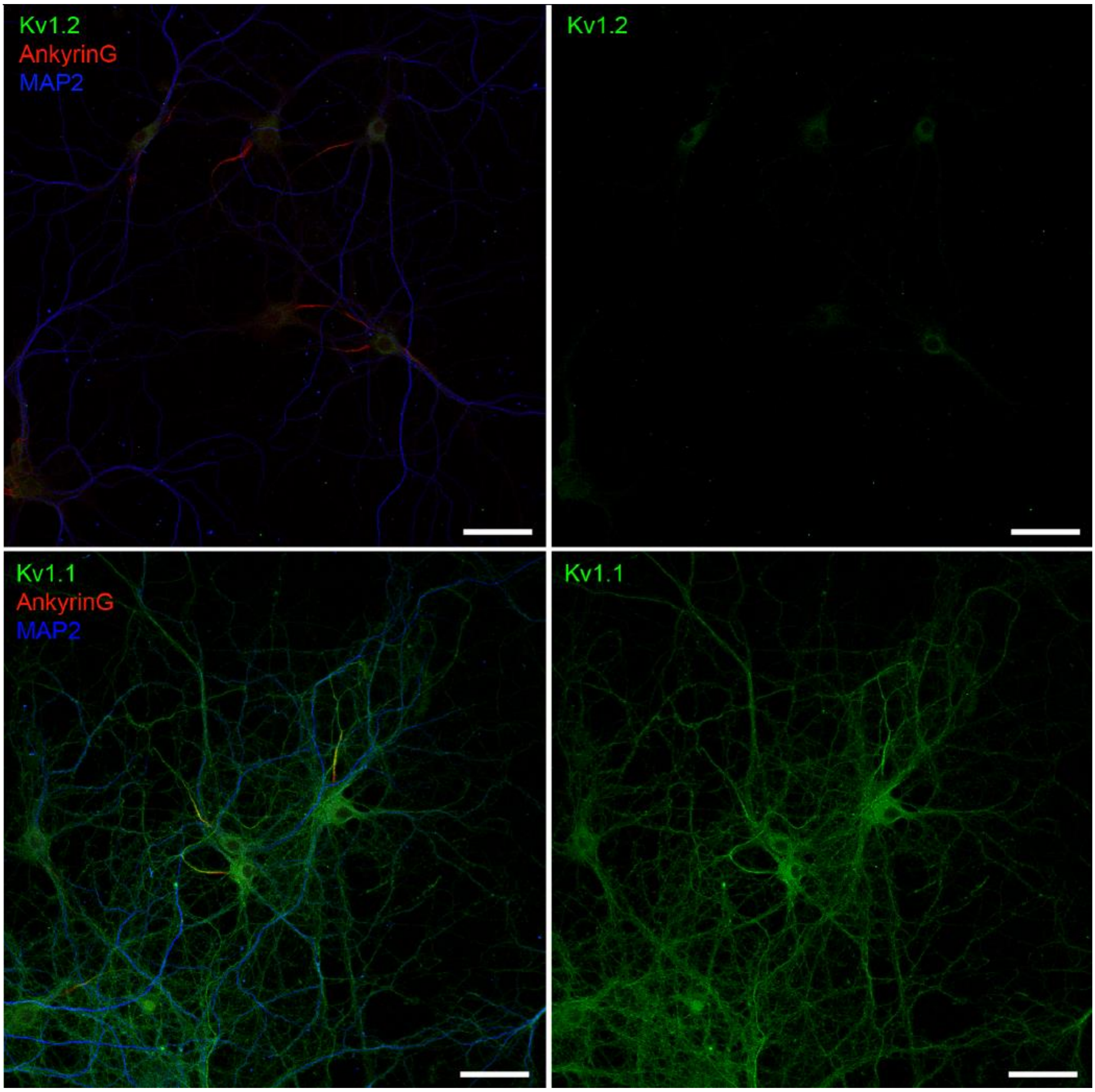

Supplementary Figure 1. Comparison of immunohistochemical staining of Kv1.2 and Kv1.1 channels in 14 DIV cultured hippocampal neurons. Upper left panel, neurons immunolabeled with Kv1.2 (green), AnkyrinG (red) and the somatodendritic marker MAP2 (blue). Upper right panel, immunolabeling of the same neurons with Kv1.2 alone. Lower left panel, neurons immunolabeled with Kv1.1 (green), AnkyrinG (red) and MAP2 (blue). Lower right panel, immunolabeling with Kv1.1. Scale bars: 50 µm.

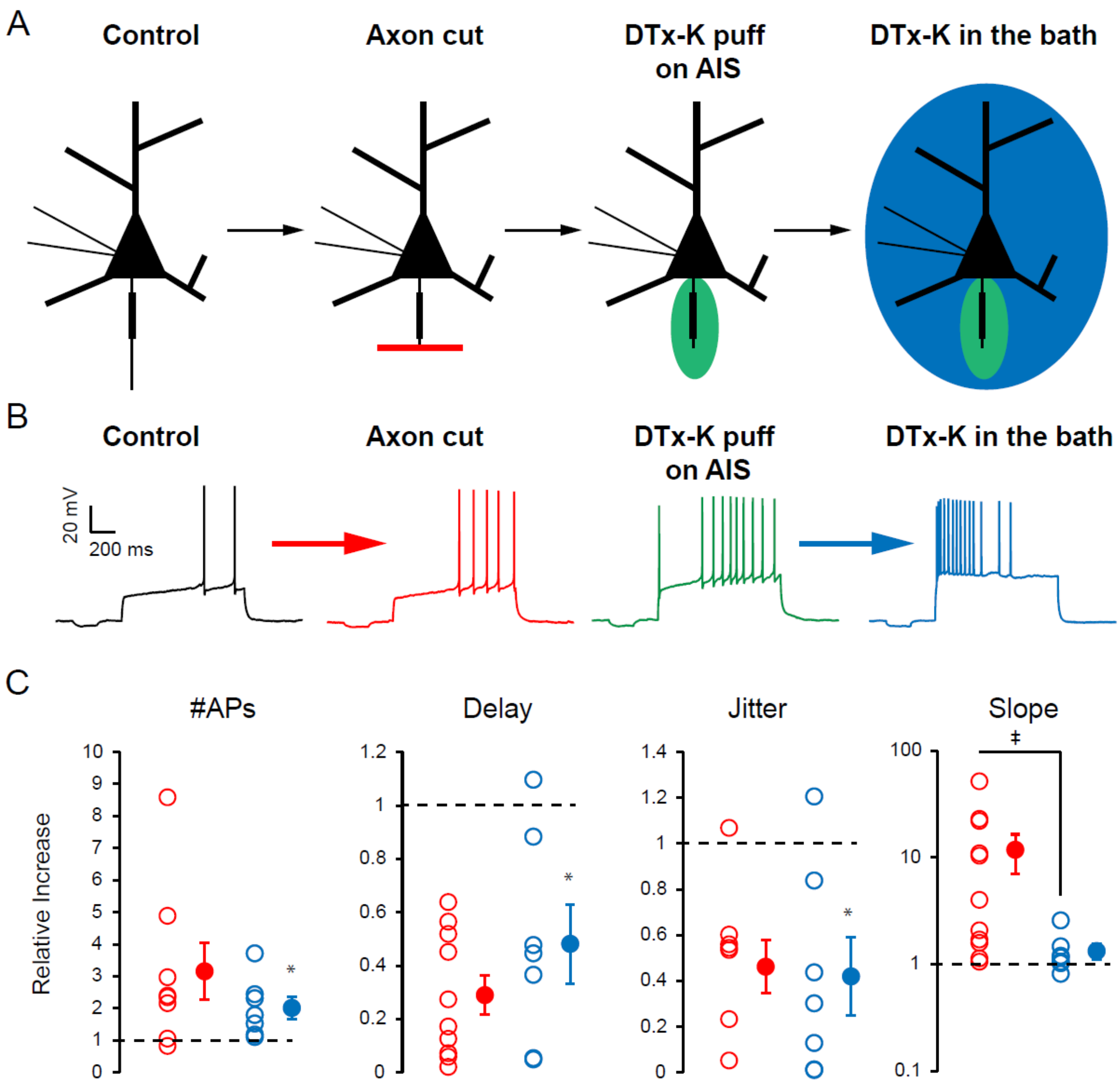

Supplementary Figure 2. A, experimental procedure for comparing the role of Kv1.1 channels in the axon proper to those located in the somato-dendritic compartment. From left to right: we started by sectioning the axon close to the AIS, then puffed DTx-K on the AIS and finally applied DTx-K in the bath. **B**, Evolution of intrinsic excitability measured in the cell all along the different steps of the experiment. Red arrow: comparison of the "*Axon cut*" condition to the "*Control*" condition in order to measure the effect of axonal Kv1.1 channels on intrinsic excitability. Blue arrow: comparison of the "*DTx-K in the bath*" condition to the "*DTx-K puff on AIS*" condition in order to measure the effect of somato-dendritic Kv1.1 channels on intrinsic excitability. **C**, comparison of the inhibition of axonal (red) and somato-dendritic (blue) Kv1.1 channels on excitability properties. Exception made for the slope before the first AP ($p<0.05$, Mann-Whitney test), the two effects are not statistically different ($p>0.05$, Mann-Whitney test). Asterisk: significant effect of applying DTx-K in the bath (Wilcoxon test). Dagger: Significant difference in the inhibition of axonal (red) versus somato-dendritic (blue) Kv1.1 channels on intrinsic excitability (Mann-Whitney test).
